# Heart rate variability during a cognitive reappraisal task in female patients with borderline personality disorder: the role of comorbid posttraumatic stress disorder and dissociation

**DOI:** 10.1017/S0033291718002489

**Published:** 2018-09-10

**Authors:** Annegret Krause-Utz, Julia-Caroline Walther, Stefanie Lis, Christian Schmahl, Martin Bohus

**Affiliations:** 1 Institute of Clinical Psychology, Leiden University; Leiden Institute for Brain and Cognition; Leiden, The Netherlands; 2 Institute of Psychiatric and Psychosomatic Psychotherapy, Central Institute of Mental Health, Mannheim; Medical Faculty, University of Heidelberg, Mannheim, Germany; 3 Department of Psychosomatic Medicine and Psychotherapy, Central Institute of Mental Health, Mannheim; Medical Faculty, University of Heidelberg, Mannheim, Germany; 4 Department of Psychiatry, Schulich School of Medicine and Dentistry, Western University, London, Ontario, Canada

**Keywords:** Borderline personality disorder, dissociation, emotion regulation, heart rate variability, posttraumatic stress disorder

## Abstract

**Background:**

Emotion dysregulation is a core feature of borderline personality disorder (BPD), which often co-occurs with posttraumatic stress disorder (PTSD). Difficulties in emotion regulation (ER) have been linked to lower high-frequency heart rate variability (HF-HRV), a measure of autonomous nervous system functioning. However, previous research on vagally-mediated heart rate in BPD revealed heterogeneous findings and the effects of comorbid PTSD and dissociation on HF-HRV are not yet completely understood. This study aim to investigate HF-HRV during resting-state and an ER task in female BPD patients with comorbid PTSD (BPD + PTSD), patients without this comorbidity (BPD), and healthy controls (HC).

**Methods:**

57 BPD patients (BPD: *n* = 37, BPD + PTSD: *n* = 20) and 27 HC performed an ER task with neutral, positive, and negative images. Participants were instructed to either attend these pictures or to down-regulate their upcoming emotions using cognitive reappraisal. Subjective arousal and wellbeing, self-reported dissociation, and electrocardiogram data were assessed.

**Results:**

Independent of ER instruction and picture valence, both patient groups (BPD and BPD + PTSD) reported higher subjective arousal and lower wellbeing; patients with BPD + PTSD further exhibited significantly lower HF-HRV compared with the other groups. Higher self-reported state dissociation predicted higher HF-HRV during down-regulating *v.* attending negative pictures in BPD + PTSD.

**Conclusions:**

Findings suggest increased emotional reactivity to negative, positive, and neutral pictures, but do not provide evidence for deficits in instructed ER in BPD. Reduced HF-HRV appears to be particularly linked to comorbid PTSD, while dissociation may underlie attempts to increase ER and HF-HRV in BPD patients with this comorbidity.

## Introduction

Borderline personality disorder (BPD) is a severe mental disorder, characterized by a marked instability in affect, self-image, and interpersonal relationships (APA, 2013). A complex interplay of genetic, neurobiological predispositions and stressful life events is assumed to underlie its development (Lieb *et al*., 2004; Crowell *et al*., 2009; Schmahl *et al*., 2014). Traumatic stress, including severe childhood maltreatment, is highly prevalent in BPD (Battle *et al*., 2004; Ball and Links, 2009), 30–80% of patients suffer from comorbid posttraumatic stress disorder (PTSD) (Zanarini *et al*., 1998; Zimmerman and Mattia, 1999; Grant *et al*., 2008; Pagura *et al*., 2010; Westphal *et al*., 2013; Frías and Palma, 2015). Although traumatic experiences are neither necessary nor sufficient for the development of BPD, (post)traumatic stress seems to aggravate symptoms, such as emotion dysregulation and dissociation (Scheiderer *et al*., 2015; Cackowski *et al*., 2016).

Emotion dysregulation is a core feature of BPD, involving increased sensitivity and reactivity to emotional stimuli, chronic affective instability, and maladaptive stress coping, e.g. non-suicidal self-harm (Crowell *et al*., 2009; Carpenter and Trull, 2013; Santangelo *et al*., 2017). There is ample evidence for self-reported difficulties in emotion regulation (ER) (Rosenthal *et al*., 2008; Glenn and Klonsky, 2009; Carpenter and Trull, 2013) and altered reactivity in fronto-limbic brain regions implicated in emotional processing and regulation (Krause-Utz *et al*., 2014; Schmahl *et al*., 2014; van Zutphen *et al*., 2015; Schulze *et al*., 2016) in BPD patients compared with healthy controls (HC). However, studies using psychophysiological markers, such as heart rate variability (HRV), revealed mixed findings (Cavazzi and Becerra, 2014).

HRV refers to the variation in time intervals between heartbeats (beat-to-beat intervals). Vagally-mediated HR or vagal tone, i.e. parasympathetic activity exerted by the vagal nerve, is involved in the synchronization of respiratory and cardiovascular processes and assumed to play a crucial role in the flexible adaptation to stressful environmental demands, including ER (Grossman and Taylor, 2007). Respiratory sinus arrhythmia (RSA) and high-frequency HRV (HF-HRV, in the 0.15–0.40 Hz range) are established measures used to quantify vagal tone and to evaluate activity of the parasympathetic nervous system relative to the sympathetic nervous system (Appelhans *et al*., 2006; Grossman and Taylor, 2007; Thayer *et al*., 2012; Williams *et al*., 2015; Verkuil *et al*., 2016).

Reduced HRV has been linked to detrimental long-term effects on physical and mental health, including increased risk for immune dysfunction, inflammation, cardiovascular diseases (Liao *et al*., 2002; Kemp and Quintana, 2013), major depression, anxiety (Kemp *et al*., 2012; Chalmers *et al*., 2014; Koenig *et al*., 2016*a*), and PTSD (Pechtel and Pizzagalli, 2011; Sammito *et al*., 2015; Meyer *et al*., 2016).

In BPD, previous research on HRV revealed inconsistencies, which may partly relate to differences in study design (resting-state *v.* experimental studies) and sample characteristics (e.g. medication status, gender, trauma history, and comorbidities). As to resting-state HRV, a recent meta-analysis points to lower vagally-mediated heart rate in BPD patients compared with HC (Koenig *et al*., 2016*b*). The majority of studies included in this analysis involved medicated samples (Kuo and Linehan, 2009; Dixon-Gordon *et al*., 2011; Gratz *et al*., 2013) or patients with unclear medication status (Weinberg *et al*., 2009), which may affect HRV (Ebner-Priemer *et al*., 2007), studies including un-medicated BPD samples found no alterations in baseline HRV (Austin *et al*., 2007; Reitz *et al*., 2015; Meyer *et al*., 2016). In the study by Meyer and colleagues (2016), during a 5-min resting-state electrocardiogram recording, a measure of HRV (RMSSD) was the lowest in un-medicated patients with PTSD, followed by un-medicated BPD patients in remission, patients with current BPD, and HC, while the latter groups did not differ significantly. Across groups, higher childhood trauma severity predicted lower HRV, suggesting a complex interplay of traumatic experiences and autonomic nervous system functioning.

Studies investigating *emotional reactivity* revealed similar inconsistencies: Austin *et al*. (2007) found no baseline differences but decreases in RSA (vagally-mediated HR), during an experimental condition with emotional film clips in BPD patients compared with HC. In contrast, Kuo and Linehan (2009) observed reduced baseline RSA and increased RSA during sad film clips in BPD.

During a stressful task, individuals with BPD features showed decreased baseline RSA and increased sympathetic activity (Weinberg *et al*., 2009). In other studies using experimental stressors, reduced HRV was only observed in BPD patients with comorbid avoidant personality (Gratz *et al*., 2013) and during a painful stimulation (incision into the forearm) (Reitz *et al*., 2015). In the study by Gratz and colleagues (2013), those with BPD and comorbid avoidant personality disorder showed greater decreases in HF-HRV in response to a stressor, suggesting poor ER.

In a comprehensive assessment of emotional processing in BPD, Kuo and colleagues (2016) investigated HRV during baseline, *emotional reactivity* (passive viewing of negative *v.* neutral images), and *ER* (mindfulness-based awareness *v.* distraction-based strategies). Patients with BPD showed reduced baseline RSA but did not differ from HC during the ER task, indicating a similar adaptation to distressing conditions. Similarly, other data suggest that individuals with BPD do not significantly differ in implementing ER skills (Metcalfe *et al*., 2015; Fitzpatrick and Kuo, 2016). Moreover, and unlike HC, they showed increased HRV when instructed to accept (*v.* suppress) emotional experiences during social rejection (Dixon-Gordon *et al*., 2017). Svaldi and colleagues (2012) found increases in HF-HRV from baseline to instructed ER, independent of strategy (accept/suppress emotions during a sadness-inducing film clip). All patients in this study had a lifetime diagnosis of PTSD, which might influence HF-HRV.

The above-mentioned studies mainly used negative (*v.* neutral) stimuli, while a growing body of literature in BPD suggests that positive stimuli are perceived as similarly disturbing (Rüsch *et al*., 2007; Sieswerda *et al*., 2007; Hagenhoff *et al*., 2013; Thome *et al*., 2015). Moreover, potential effects of dissociation on HF-HRV in BPD remain unclear. Stress-related dissociative states, such as depersonalization, derealization, and numbing are a core symptom of BPD, occurring in 75–80% of patients (APA, 2013). Research in the dissociative subtype of PTSD and depersonalization suggests that dissociation may be a form of emotion over-modulation, promoting a dampening of stressful (trauma-related) emotions, probably at the cost of other mental resources crucial to goal-directed behavior, such as learning and memory (Sierra and Berrios, 1998; Lanius *et al*., 2010). Dissociation was found to dampen (reduce/attenuate) skin conductance responses (SCR) (Ebner-Priemer *et al*., 2005, 2009; Barnow *et al*., 2012; Fitzpatrick and Kuo, 2015) and limbic reactivity (Ebner-Priemer *et al*., 2005; Barnow *et al*., 2012; Fitzpatrick and Kuo, 2015), while impairing learning and memory (Ebner-Priemer *et al*., 2009; Krause-Utz *et al*., 2018) in BPD. However, studies investigating effects of dissociation on HF-HRV during emotional challenge are still scarce. To our knowledge, only one study in BPD so far reported effects of dissociation on RSA during *emotional recovery* (Fitzpatrick and Kuo, 2015). In this study, after the induction of anger (but not sadness or fear) a decrease in RSA was found, which was not specific for BPD but also observed in generalized social anxiety disorder patients. Interestingly, higher dissociation predicted *reduced* sympathetic reactivity, suggesting *better* emotional adaptation, after induction of anger and sadness. Since this study focused on *emotional recovery*, HRV studies using *ER* tasks in BPD are still needed.

This study aim to investigate HF-HRV during baseline and its adaptation during an ER (cognitive reappraisal) task with negative, positive, and neutral stimuli in BPD patients with *v.* without comorbid PTSD and HC, examining the influence of dissociation, while controlling for medication. We expected increased subjective *emotional reactivity* (higher arousal, lower wellbeing) and decreased *ER* (lower decrease/increase of arousal/wellbeing when instructed to down-regulate *v.* attend emotional pictures) in both BPD groups compared with HC. Based on previous findings (Meyer *et al*., 2016), HF-HRV were expected to be the lowest in patients with comorbid PTSD, followed by BPD patients without PTSD, and HC.

## Methods

### Participants

Initially, 68 women with BPD according to Diagnostic and Statistical Manual of Mental Disorders (DSM)-IV (American Psychiatric Association, APA, 2000), including 37 patients without PTSD and 31 patients with comorbid PTSD (BPD + PTSD), and 28 female HC participated. Inclusion criteria were ability to understand/give informed consent, aged 18–55, and female gender. Since the majority of BPD patients are female (APA, 2013) and gender may have a confounding effect on HRV (Verkuil *et al*., 2015), we included only women. Exclusion criteria for patients were lifetime history of bipolar disorder, psychotic disorder, acute life-threatening suicidal crisis, mental deficiency, and severe organic disorder. Exclusion criterion for HC was lifetime history of psychiatric disorders and severe organic disorders. Psychotropic medication was no exclusion criterion but controlled for in the analysis and matched between the two patient groups (see Table 1).

**Table 1.** Demographic and clinical characteristics of patients with BPD + PTSD, patients without comorbid PTSD (BPD), and HC

	BPD + PTSD (*n* = 20)	BPD (*n* = 37)	HC (*n* = 27)	Group statistics
*Age* [years]	34.16 ± 9.92	31.28 ± 10.28	28.88 ± 6.39	*F*_(2,77)_ = 1.85, *p* = 0.164
*Years of education*				
9 years	*n* = 4 (15%)	*n* = 4 (11%)	*n* = 1 (4%)	
10 years	*n* = 7 (35%)	*n* = 16 (43%)	*n* = 8 (39%)	χ^2^ = 3.78, *p* = 0.437
12–13 years	*n* = 10 (50%)	*n* = 17 (46%)	*n* = 18 (67%)	
*MWT-B* (*intelligence*)	29.79 ± 3.43	30.11 ± 3.49	29.88 ± 3.00	*F*_(2,77)_ = 0.07, *p* = 0.933
*DSS-4* (*state dissociation*)				
Baseline	1.55 ± 1.53^a^	1.32 ± 1.51^a^	0.00 ± 0.00^b^	*F*_(2,77)_ = 11.77, *p* < 0.0001
Before task	2.80 ± 2.39^a^	1.66 ± 1.85^a^	0.00 ± 0.00^b^	*F*_(2,77)_ = 16.53, *p* < 0.0001
After task	2.89 ± 2.70^a^	2.07 ± 2.18^a^	0.00 ± 0.00^b^	*F*_(2,77)_ = 13.14, *p* < 0.0001
*Arousal ratings*				
Baseline	4.38 ± 2.01^a^	4.89 ± 1.78^a^	2.66 ± 1.43^b^	*F*_(2,77)_ = 13.18, *p* < 0.0001
Before task	6.91 ± 2.31^a^	6.10 ± 1.52^a^	3.73 ± 2.01^b^	*F*_(2,77)_ = 19.22, *p* < 0.0001
After task	6.64 ± 1.94^a^	6.34 ± 1.91^a^	3.61 ± 1.78^b^	*F*_(2,77)_ = 11.25, *p* < 0.0001
*BSL-23 mean* (*BPD symptom severity*)	2.24 ± 0.14^a^	1.83 ± 0.10^a^	0.19 ± 0.72^b^	*F*_(2,77)_ = 73.65, *p* < 0.0001
*DES* (*trait dissociation*)	31.75 ± 2.38^a^	23.60 ± 1.73^b^	5.65 ± 4.09^c^	*F*_(2,77)_ = 37.80, *p* < 0.0001
*DERS* (*ER*)*				
Nonacceptance	23.00 ± 1.24^a^	20.90 ± 0.90^a^	10.85 ± 4.83^b^	*F*_(2,77)_ = 35.43, *p* < 0.0001
Goals	21.74 ± 1.16^a^	20.81 ± 0.63^a^	11.47 ± 4.55^b^	*F*_(2,77)_ = 55.41, *p* < 0.0001
Impulse	21.11 ± 1.56^a^	18.28 ± 0.84^a^	9.26 ± 3.10^b^	*F*_(2,77)_ = 36.70, *p* < 0.0001
Awareness	23.37 ± 1.13^a^	21.85 ± 0.82^a^	14.48 ± 5.86^b^	*F*_(2,77)_ = 22.24, *p* < 0.0001
Strategies	31.21 ± 1.32^a^	27.92 ± 0.96^a^	12.11 ± 4.71^b^	*F*_(2,77)_ = 76.49, *p* < 0.0001
Clarity	31.21 ± 1.32^a^	27.92 ± 0.96^a^	8.07 ± 3.42^b^	*F*_(2,77)_ = 55.00, *p* < 0.0001
*ERQ* (*regulation strategies*)				
Cognitive reappraisal	3.29 ± 0.24^a^	3.59 ± 0.18^a^	4.72 ± 1.06^b^	*F*_(2,77)_ = 13.08, *p* < 0.0001
Suppression	4.15 ± 0.29^a^	3.94 ± 0.21^a^	2.58 ± 1.03^b^	*F*_(2,77)_ = 17.09, *p* < 0.0001
*BDI-II* (*depression*)	36.42 ± 9.73^a^	29.67 ± 12.04^a^	5.00 ± 5.42^b^	*F*_(2,77)_ = 68.03, *p* < 0.0001
*STAI* (*state anxiety*)	48.60 ± 9.93^a^	54.32 ± 10.96^a^	29.94 ± 4.53^b^	*F*_(2,77)_ = 49.22, *p* < 0.0001
*CTQ* (*Childhood trauma*)**	82.86 ± 17.86^a^	56.79 ± 13.74^b^	32.15 ± 11.59^c^	*F*_(2,77)_ = 63.02, *p* < 0.0001

Values are presented in means ± s.d. or frequencies (*n*) and percentages (%).

BDI-II, Beck Depression Inventory-II; BSL-23, Borderline Symptom List 23; DERS, Difficulties in Emotion Regulation Scale (*higher scores indicate more difficulties in ER); Nonacceptance, nonacceptance of emotions, Goals, difficulties in goal directed behavior, Impulse, impulse control difficulties, Awareness, lack of emotional awareness, Strategies, limited access to strategies, Clarity, lack of emotional clarity; DES, Dissociative Experience Scale; DSS-4, Dissociation Stress Scale 4; ERQ, Emotion regulation questionnaire; MWT-B, Mehrfachwortschatztest; STAI, State Anxiety Inventory, **CTQ scores were available in *n* = 19 BPD and *n* = 14 BPD + PTSD. Groups with different superscripts (a, b, c) differ significantly (*p* < 0.05).

Patients were recruited through existing databases within the Clinical Research Unit 256 (Schmahl *et al*., 2014) and advertisements in newspapers or internet platforms within a larger longitudinal training study (reported elsewhere). HC were recruited from a pool of healthy subjects that previously agreed to participate in future studies. All participants underwent standardized diagnostic interviews by trained clinical psychologists, including the Structured Interview for DSM-IV (SCID-I) (First *et al*., 1997) and International Personality Disorder Examination (IPDE) (Loranger, 1999) to assess BPD or to assure that diagnostic criteria for BPD were still met in patients recruited from a database. Further assessments included a test of intelligence (MWT) (Lehrl, 1989), questionnaires on BPD symptom severity (Borderline Symptom List 23, BSL-23) (Bohus *et al*., 2009), depression (Beck Depression Inventory, BDI) (Hautzinger *et al*., 2009), trait dissociation (Dissociation Experience Scale, DES) (Bernstein and Putnam, 1986), and anxiety (State Anxiety Questionnaire, STAI) (Laux *et al*., 1981). Subjective ER was assessed by the Difficulties in ER Scale (DERS) (Gratz and Roemer, 2004) and a modified version of the ER Questionnaire (ERQ) (Gross and John, 2003) (online Supplementary Material).

From 68 patients, six patients did not complete the study due to increased distress. Five BPD patients and one HC whose HRV data were contaminated by artifacts, caused by technical problems, were excluded from the final analysis. The final sample comprised 27 HC, 37 patients without PTSD (BPD), and 20 patients with comorbid PTSD (BPD + PTSD). Overall, 23 patients received psychotropic medication, approximately 40.5% per group (BPD: *n* = 15; BPD + PTSD: *n* = 8; Table 1 and online Supplementary Table S1).

There were no group differences in age, years of education, and intelligence (Table 1). As expected, both patient groups scored significantly higher on clinical measures than HC. The two patient groups did not differ significantly in BPD symptom severity, depressive symptoms, difficulties in ER, use of cognitive reappraisal or suppression (Table 1), and comorbidities other than PTSD (Table 2). Patients in the BPD + PTSD group reported higher trait dissociation (DES) and more severe childhood trauma (CTQ) (Table 1).

**Table 2.** List of medications and comorbidities in patients with BPD + PTSD and BPD patients without comorbid PTSD (BPD)

	BPD + PTSD (*n* = 20)	BPD (*n* = 37)	Group comparisons
*Medication*	*n* = 8 (40%)	*n* = 15 (40.5%)	χ^2^ = 0.02, *p* = 0.903
SSRI (citalopram, escitalopram, fluoxetine, sertraline)	*n* = 5	*n* = 11	χ^2^ = 0.29, *p* = 0.591
Tricyclica (trimipramine)	*n* = 1	*n* = 0	χ^2^ = 1.96, *p* = 0.161
SARI (trazodone)	*n* = 1	*n* = 1	χ^2^ = 0.22, *p* = 0.636
SNRI (venlafaxine)	*n* = 3	*n* = 3	χ^2^ = 0.83, *p* = 0.363
Mood stabilizer (lamotrigine)	*n* = 0	*n* = 1	χ^2^ = 0.58, *p* = 0.455
Buproprion	*n* = 0	*n* = 2	χ^2^ = 1.68, *p* = 0.280
Mirtazipine	*n* = 0	*n* = 1	χ^2^ = 0.58, *p* = 0.455
Atypical antipsychotics			
(Quetiapine, Risperidone)	*n* = 2	*n* = 5	χ^2^ = 0.17, *p* = 0.679
Typical antipsychotics (Perazine)	*n* = 0	*n* = 1	χ^2^ = 0.58, *p* = 0.455
*Comorbidities*			
*Major depression current*	*n* = 7	*n* = 14	χ^2^ = 0.29, *p* = 0.592
*Major depression lifetime*	*n* = 17	*n* = 31	χ^2^ = 1.17, *p* = 0.280
*Dysthymia current*	*n* = 3	*n* = 8	χ^2^ = 1.20, *p* = 0.274
*Dysthymia lifetime*	*n* = 3	*n* = 8	χ^2^ = 1.20, *p* = 0.274
*Alcohol abuse lifetime*	*n* = 5	*n* = 8	χ^2^ = 1.41, *p* = 0.493
*Substance abuse lifetime*	*n* = 6	*n* = 9	χ^2^ = 0.22, *p* = 0.642
*Substance dependence lifetime*	*n* = 6	*n* = 10	χ^2^ = 0.06, *p* = 0.812
*Panic disorder current*	*n* = 6	*n* = 3	χ^2^ = 2.66, *p* = 0.103
*Panic disorder lifetime*	*n* = 6	*n* = 3	χ^2^ = 2.66, *p* = 0.103
*Social phobia current*	*n* = 7	*n* = 6	χ^2^ = 0.73, *p* = 0.393
*Social phobia lifetime*	*n* = 7	*n* = 6	χ^2^ = 0.73, *p* = 0.393
*Specific phobia current*	*n* = 3	*n* = 2	χ^2^ = 0.66, *p* = 0.416
*Specific phobia lifetime*	*n* = 3	*n* = 2	χ^2^ = 0.66, *p* = 0.416
*OCD current*	*n* = 2	*n* = 2	χ^2^ = 0.11, *p* = 0.735
*OCD lifetime*	*n* = 2	*n* = 2	χ^2^ = 0.11, *p* = 0.735
*GAD current*	*n* = 2	*n* = 1	χ^2^ = 1.44, *p* = 0.486
*GAD lifetime*	*n* = 2	*n* = 1	χ^2^ = 1.44, *p* = 0.486
*Pain disorder current*	*n* = 0	*n* = 1	χ^2^ = 1.54, *p* = 0.464
*Pain disorder lifetime*	*n* = 0	*n* = 1	χ^2^ = 1.54, *p* = 0.464
*Anorexia current*	*n* = 0	*n* = 1	χ^2^ = 0.70, *p* = 0.403
*Anorexia lifetime*	*n* = 5	*n* = 6	χ^2^ = 0.12, *p* = 0.725
*Bulimia current*	*n* = 2	*n* = 8	χ^2^ = 2.22, *p* = 0.136
*Bulimia lifetime*	*n* = 7	*n* = 7	χ^2^ = 0.25, *p* = 0.618

Values are presented in means ± s.d. or frequencies (*n*) and percentages (%).

GAD, generalized anxiety disorder; OCD, obsessive compulsive disorder; SARI, serotonin antagonist and reuptake inhibitor; SSRI, selective serotonin reuptake inhibitors; SNRI, serotonin and norepinephrine reuptake inhibitor.

### Material

The *ER task* was an adapted cognitive reappraisal paradigm (Ochsner *et al*., 2002; Koenigsberg *et al*., 2009; Schulze *et al*., 2011). Participants were instructed to either pay attention to the pictures without actively attempting to regulate upcoming emotions (*attend*), or to *down-regulate* upcoming emotions, e.g. reinterpret the content of pictures. Following a standardized procedure, two practice trials with examples and feedback were provided to ensure participants understood task instructions correctly. The task involved 105 pictures of three stimulus categories (42 negative, 42 positive, and 21 neutral pictures) from the International Affective Picture System (IAPS) (Lang *et al*., 2008). Based on existing norms, ‘negative’ stimuli were selected based on high arousal (mean = 6.41, s.d. = 2.19), and low valence ratings (1.79 ± 1.18), ‘positive’ pictures had high arousal (4.99 ± 2.38) and high valence ratings (8.02 ± 1.26), and ‘neutral’ pictures had low arousal (3.19 ± 1.93) and moderate valence ratings (5.23 ± 1.23) (see online Supplementary Material). Using a randomized picture block design, 15 picture blocks of seven pictures were presented, starting with an instruction (*attend* or *downregulate*) (4s), followed by seven pictures (6s). The five task conditions (neutral_attend, positive_attend, negative_attend, positive_downregulate, and negative_downregulate) were presented threefold each (online Supplementary Fig. S1). Order of picture blocks and instructions was randomized and balanced per condition resulting in 12 pseudorandom sequences. Before and after each picture block, participants rated their subjective arousal (between 1 = ‘not at all aroused’ and 10 = ‘extremely aroused’) and emotional well-being (−10 = ‘not at all well’, +10 = ‘extremely well’) on a visual analog scale (Self-Assessment Manikin, SAM) (Bradley and Lang, 1994). At baseline, before and after the task, participants completed the Dissociation Stress Scale 4 (DSS-4) (Stiglmayr *et al*., 2009), i.e. four items on acute dissociative states and one item on aversive inner tension (between ‘0 = not at all’ and ‘10 = extremely’). Presentation software (Neurobehavioral Systems, Inc., Berkeley, USA) was used to present stimuli via a 17.3″-monitor located on a table in the laboratory.

### Procedure

The study was approved by the local ethics committee of Heidelberg University. All participants were informed about the study. After giving written informed consent, they underwent clinical assessments. The experiment was conducted in a laboratory at the Institute for Psychiatric and Psychosomatic Psychotherapy of the Central Institute of Mental Health (CIMH) in Mannheim. Electrocardiogram (EKG) data were continuously obtained during the experiment, according to guidelines of the Task Force of the European Society of Cardiology and the North American Society of Pacing and Electrophysiology (Malik, 1996) using devices of the Biosemi Active Two system with a sampling rate of 1024 Hz (Biosemi, Amsterdam, Netherlands, ActiView software; http://www.biosemi.com). Two Ag/AgCl electrodes were placed under the right clavicle and on the left side under the lowest rib. Participants were instructed to sit still, while a 5-min baseline assessment of HRV was conducted. To create a relaxing atmosphere, a soundless 5-min nature film was presented in the background. Then, the experimenter left the room and participants performed the ER task (13 min). Participants further performed a working memory task (reported elsewhere). At the end, subjects were debriefed, thanked, and paid for their participation (12€/h).

### Statistical analysis

Using software IBM SPSS Statistics Version 23, group and task effects on the dependent variables (DVs) were evaluated using separate analyses of variance (ANOVA), described below. Assumptions of normality, linearity, homoscedasticity, and sphericity (Kolmogorov–Smirnov tests, visual inspection of graphic plots, Mauchly's test with Greenhouse–Geisser-corrections) and equality of variances (Levene's test) were checked. Significant ANOVA effects were followed up using Tukey's HSD tests for multiple comparisons, independent-sample *t* tests, and paired *t* tests, respectively. Significance level for all analyses was *p* = 0.05, two-tailed. For significant effects, partial *η*^2^†^1^ or Cohen's *d*^2^ are reported (Cohen, 1988). To illustrate changes in DVs for ‘*down-regulate*’ *v.* ‘*attend*’, contrasts between these ER conditions were calculated for positive and negative pictures (regulate_minus_attend_negative and regulate_minus_attend_positive, respectively).

#### Ratings

Ratings of arousal and emotional well-being were analyzed using separate repeated measures ANOVAs (rm-ANOVAs). To evaluate *emotional reactivity*, a 3 × 3 repeated rm-ANOVA with group as between-subject factor and valence of pictures in the passive viewing condition (negative_attend, positive_attend, and neutral_attend) as within-factor was performed. To analyze *ER*, a 3 × 2 × 2 rm-ANOVA with instruction (attend, downregulate) and valence (negative, positive) as within-factors was conducted. No 3 × 2 × 3 rm-ANOVA could be performed, as there was only one instruction (*attend*) for neutral pictures.

#### HF-HRV

EKG data were preprocessed using software Kubios HRV [Finland, http://kubios.uef.fi/ (Tarvainen *et al*., 2014)]. Recorded data were visually inspected for false or undetected R-waves, data with technical or physiological artifacts were excluded. Spectral analysis was used to calculate high frequency variations in beat-to-beat intervals (0.15–0.40 Hz). As recommended by current guidelines (Malik, 1996) and to improve robustness of data, HF-HRV measures of the 15 ER task blocks were averaged for each condition, resulting in five DVs (neutral_attend, positive_attend, negative_attend, positive_downregulate, and negative_downregulate). Data were analyzed using an autoregressive (AR) model, which is independent of signal length (Parati *et al*., 1995; Di Simplicio *et al*., 2012). HF-HRV measures are reported in normative units, evaluating the relative percentage (%) of parasympathic to sympathic activity, which is thought to reduce noise stemming from artifacts (Malik, 1996). Analyses were also performed for absolute values. Group differences in baseline HF-HRV were tested using a univariate ANOVA. HF-HRV during the ER task was evaluated equivalent to the above-mentioned analysis of ratings (*emotional reactivity:* 3 × 3 rm-ANOVA, *ER*: 3 × 2 × 2 rm-ANOVA).

*Post-hoc analyses*: Since 21 patients had current major depression, which may affect HF-HRV (Kemp *et al*., 2012; Koenig *et al*., 2016*a*), additional HF-HRV analyses excluding patients with this comorbidity were performed.

*Medication:* In separate rm-ANCOVAs, medication status (no medication, antidepressants, and antipsychotics) was included as statistical covariate to test for effects on HF-HRV results. Additionally, all HF-HRV analyses were repeated excluding medicated patients.

*Dissociation:* Changes in dissociation over the course of the experiment were evaluated using a 2 × 3 rm-ANOVA with group (BPD + PTSD, BPD) as between-subject factor and time (baseline, before task, and after task) as within-subject factor.

To test whether dissociation predicted emotional reactivity/regulation, multiple regression analyses were performed, with mean DSS-4 scores at baseline, before and after task as predictors and HF-HRV values for neutral_attend, positive_attend, negative_attend (*emotional reactivity*), regulate_minus_attend_negative, and regulate_minus_attend_positive (*emotional regulation*), respectively as DVs. It was checked whether results changed after adding arousal, DERS, ERQ, BSL, BDI, and STAI scores as covariates. Regression analyses were performed for BPD and BPD + PTSD separately, adjusting significance levels for the number of groups (*p* ⩽ 0.025, two-tailed).

## Results

### Subjective ratings

Results of the rm-ANOVAs and post-hoc tests are reported in Table 3.

**Table 3.** Results of the ANOVA and post-hoc group tests for subjective ratings

Emotional reactivity: 3 × 3 rm-ANOVA (*F*_(df)_, *p*, *η*_p_^2^) post-hoc Tukey's tests and paired *t* tests				
*Arousal*				
Group	*F*_(2,81)_ = 7.01	*p* = 0.002	*η*_p_^2^ = 0.15	BPD *v*. HC: *p* = 0.002, *d* = 0.92 BPD *+* PTSD *v*. HC: *p* = 0.027, *d* = 0.89 BPD *v*. BPD *+* PTSD: *p* = 0.899
Valence	*F*_(2,80)_ = 103.40	*p* < 0.0001	*η*_p_^2^ = 0.72	Negative *v*. neutral: *t*_(83)_ = 13.61, *p* < 0.0001, *d* = 1.64 Negative *v*. positive: *t*_(83)_ = 14.87, *p* < 0.0001, *d* = 1.91
Group × valence	*F*_(2,81)_ = 0.14	*p* = 0.969		
*Well-being*				
Group	*F*_(2,81)_ = 3.43	*p* = 0.037	*η*_p_^2^ = 0.08	BPD *v*. HC: *p* = 0.028, *d* = 0.71 BPD *+* PTSD *v*. HC: *p* = 0.369 BPD *v*. BPD *+* PTSD: *p* = 0.612
Valence	*F*_(2,80)_ = 224.56	*p* < 0.0001	*η*_p_^2^ = 0.85	Negative *v*. neutral: *t*_(83)_ = 17.35, *p* < 0.0001, *d* = 2.76 Negative *v*. positive: *t*_(83)_ = 22.30, *p* < 0.0001, *d* = 3.91 Positive *v.* neutral*: t*_(83)_ = 11.87, *p* < 0.0001, *d* = 1.43
Group × valence	*F*_(2,81)_ = 0.36	*p* = 0.835		
*Emotional regulation: 3* *×* *2* *×* *3 rm-ANOVA* (*F*_(*df*)_, *p, η*_*p*_^2^) *post-hoc Tukey's tests and paired t tests*				
*Arousal*				
Group	*F*_(2,81)_ = 6.36	*p* = 0.003	*η*_*p*_^2^ = 0.14	BPD *v*. HC: *p* = 0.003, *d* = 0.91 BPD *+* PTSD: *p* = 0.039, *d* = 0.90 BPD *v*. BPD *+* PTSD: *p* = 0.903
Valence	*F*_(1,81)_ = 164.12	*p* < 0.0001	*η*_p_^2^ = 0.67	Negative *v*. positive: *t*_(83)_ = 13.36, *p* < 0.0001, *d* = 1.59
Instruction	*F*_(1,81)_ = 3.86	*p* = 0.053	*η*_p_^2^ = 0.05	
Group × valence	*F*_(1,81)_ = 0.11	*p* = 0.892		
Group × instruction	*F*_(1,81)_ = 0.15	*p* = 0.857		
Valence × instruction	*F*_(1,81)_ = 36.88	*p* < 0.0001	*η*_p_^2^ = 0.31	Negative attend *v*. regulate: *t*_(83)_ = 4.28, *p* < 0.0001, *d* = 0.28 Positive attend *v*. regulate: *t*_(83)_ = −5.70, *p* < 0.0001, *d* = 0.45
Group × valence × instruction	*F*_(2,81)_ = 0.73	*p* = 0.581		
*Well-being*				
Group	*F*_(2,81)_ = 3.77	*p* = 0.027	*η*_p_^2^ = 0.09	BPD *v*. HC: *p* = 0.025, *d* = 0.75 BPD *+* PTSD *v*. HC: *p* = 0.144, BPD *+* PTSD *v*. BPD: *p* = 0.915
Valence	*F*_(1,81)_ = 348.42	*p* < 0.0001	*η*_p_^2^ = 0.81	Negative *v*. positive: *t*_(83)_ = 19.56, *p* < 0.0001, *d* = 3.28
Instruction	*F*_(1,81)_ = 17.19	*p* < 0.0001	*η*_p_^2^ = 0.18	Attend *v*. regulate: *t*_(83)_ = 4.14, *p* < 0.0001, *d* = 0.41
Group × valence	*F*_(1,81)_ = 0.25	*p* = 0.783		
Group × instruction	*F*_(1,81)_ = 0.04	*p* = 0.964		
Valence × instruction	*F*_(1,81)_ = 98.32	*p* < 0.0001	*η*_p_^2^ = 0.55	Negative attend *v*. regulate: *t*_(83)_ = 7.11, *p* < 0.0001, *d* = 0.60 Positive attend *v*. regulate: *t*_(83)_ = 8.82, *p* < 0.0001, *d* = 0.90
Group × valence × instruction	*F*_(2,81)_ = 0.73	*p* = 0.581		

BPD + PTSD, borderline personality disorder patients with comorbid posttraumatic stress disorder (PTSD); BPD, borderline personality disorder patients without comorbid posttraumatic stress disorder (PTSD); HC, healthy controls.

#### Emotional reactivity

**Fig. 1.**
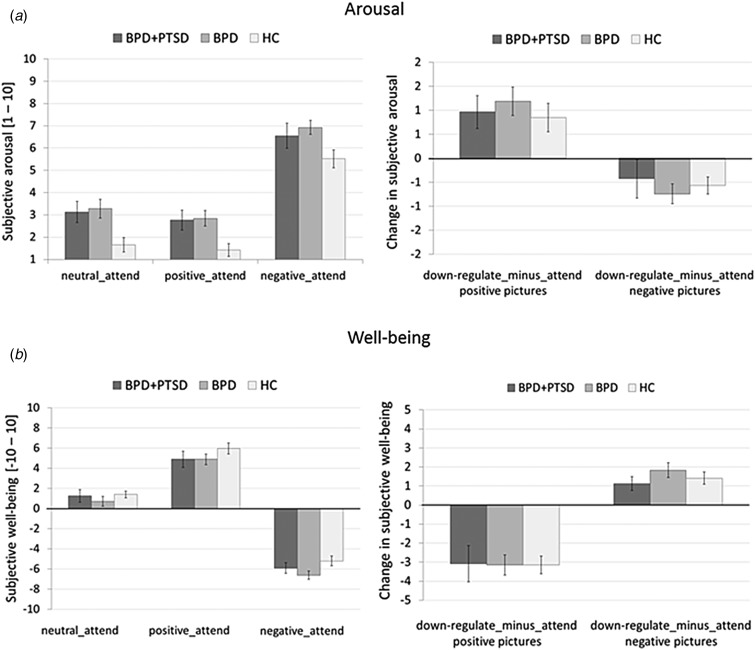
Means and standard errors of the mean of subjective ratings for arousal (*a*) and emotional well-being (*b*) in patients with BPD + PTSD, BPD patients without comorbid PTSD (BPD), and HC during the ER task. The left graphs depict arousal ratings for neutral, positive, and negative pictures in the passive viewing conditions (negative_attend, positive_attend, neutral_attend, and *emotional reactivity*). The right graphs illustrate changes in ratings for the ‘*down-regulate*’ minus ‘*attend’* condition (regulate_minus_attend_negative, regulate_minus_attend_positive, and *ER*).

*Arousal*: Arousal was higher in both BPD and BPD + PTSD compared with HC (Fig. 1*a*). This group difference was not influenced by picture valence. Valence had a significant main effect: arousal ratings were higher for negative than for neutral and positive pictures. There was no significant interaction effect.

*Emotional well-being*: A significant group effect, with lower well-being in BPD than in HC was found (Fig. 1*b*). Picture valence had a significant main effect: attending negative stimuli resulting in lower well-being compared with neutral and positive pictures. Attending positive stimuli resulted in higher well-being compared with neutral pictures. The interaction effect was not significant.

#### ER

*Arousal*: Ratings were higher in both BPD and BPD + PTSD compared with HC. This group difference was not influenced by ER instruction or picture valence. Valence influenced arousal ratings, with higher arousal for negative *v.* positive pictures, and significantly interacted with instruction: arousal ratings for negative pictures were higher in the ‘attend’ *v.* ‘down-regulate’ condition, while the opposite was found for positive pictures.

*Well-being:* Groups differed in emotional well-being, with lower ratings in BPD than HC. Picture valence influenced the ratings, with lower well-being for negative *v.* positive pictures. Instruction had a significant main effect (well-being was lower for ‘attend’ *v.* ‘down-regulate’) and significantly interacted with valence: instructions to down-regulate increased well-being for negative pictures but decreased well-being for positive pictures.

### HF-HRV

Results of the ANOVAs and post-hoc tests for HF-HRV (n.u.) are summarized in Table 4 (for absolute HF-HRV see online Supplementary Table S2 and Fig. S2).

**Table 4.** Results of the ANOVA and post-hoc group tests for HF-HRV for the whole group (HC: *n* = 27, BPD + PTSD: *n* = 20, BPD: *n* = 37)

	*F*(df)	*p*	*η*_p_^2^	Post-hoc group tests
*Baseline: univariate ANOVA*				
Group	*F*_(2,78)_ = 2.76	*p* = 0.069	*η*_p_^2^ = 0.07	BPD *+* PTSD *v*. HC: *p* = 0.061 BPD *+* PTSD *v*. BPD: *p* = 0.177 BPD *v*. HC: *p* = 0.770
*Emotional reactivity: 3* *×* *3 rm-ANOVA*				
Group	*F*_(2,81)_ = 6.42	*p* = 0.003	*η*_p_^2^ = 0.14	BPD *+* PTSD *v*. HC: *p* = 0.010, *d* = 1.00 BPD *+* PTSD *v*. BPD: *p* = 0.056, *d* = 0.62 BPD *v*. HC: *p* = 0.956
Valence	*F*_(2,80)_ = 0.68	*p* = 0.507		
Group × valence	*F*_(4158)_ = 1.24	*p* = 0.293		
*Emotional regulation: 3* *×* *2* *×* *3 rm-ANOVA*				
Group	*F*_(2,80)_ = 6.69	*p* = 0.002	*η*_p_^2^ = 0.14	BPD *+* PTSD *v*. HC: *p* = 0.012, *d* = 1.00 BPD *+* PTSD *v*. BPD: *p* = 0.022, *d* = 0.69 BPD *v*. HC: *p* = 0.999
Instruction	*F*_(1,81)_ = 3.05	*p* = 0.085	*η*_p_^2^ = 0.04	
Valence	*F*_(1,81)_ = 0.14	*p* = 0.708		
Group × instruction	*F*_(2,81)_ = 0.58	*p* = 0.564		
Group × valence	*F*_(2,81)_ = 0.51	*p* = 0.601		
Valence × instruction	*F*_(1,81)_ = 3.14	*p* = 0.080	*η*_p_^2^ = 0.04	
Group × valence × instruction	*F*_(2,81)_ = 2.15	*p* = 0.123		

BPD + PTSD, borderline personality disorder patients with comorbid posttraumatic stress disorder (PTSD); BPD, borderline personality disorder patients without comorbid posttraumatic stress disorder (PTSD); HC, healthy controls.

*Baseline:* There was a trend for a group effect, with lower HF-HRV in BPD + PTSD than in HC and BPD.

*Emotional reactivity:* The group effect was significant: BPD + PTSD showed lower HF-HRV than HC and (as a trend) BPD, independent of picture valence and ER instruction. The other effects were not significant.

**Fig. 2.**
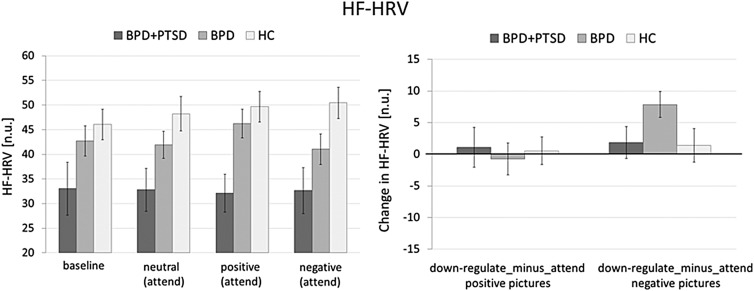
Means and standard errors of the mean of HF-HRV (normalized units) in patients with BPD + PTSD, BPD patients without comorbid PTSD (BPD), and HC during baseline conditions and the ER task. The left graph shows HF-HRV during baseline and the passive viewing conditions of the ER task (baseline, negative_attend, positive_attend, neutral_attend, and *emotional reactivity*). The right graph depicts changes in HF-HRV for the ‘*down-regulate*’ minus ‘*attend’* condition (regulate_minus_attend_negative, regulate_minus_attend_positive, and ER).

*ER:* The group effect was again significant, with significantly lower HF-HRV in BPD + PTSD compared with BPD and HC, independent of picture valence (positive, negative), and instruction (attend, down-regulate) (Fig. 2). No other effects reached statistical significance.

*Post-hoc analysis*: Results remained stable when excluding patients with major depression (online Supplementary Table S3).

*Medication*: Medication status was not a significant covariate in any analysis and did not change results (online Supplementary Table S4*a*). When repeating the analyses excluding medicated patients, baseline differences became significant, with lower HF-HRV in BPD + PTSD than in the other groups, while results for the ER task did not change (online Supplementary Table S4*b*).

*Dissociation during the ER task*: A main effect with significant increases in dissociation was found (*F*_(2,54)_ = 12.11, *p* < 0.0001, *η*^2^ = 0.31). There was no significant group effect (*F*_(1,55)_ = 2.70, *p* = 0.106, *η*^2^ = 0.05) but a significant interaction effect (*F*_(2,54)_ = 3.50, *p* = 0.037, *η*^2^ = 0.12) with stronger increases in dissociation during task compared with baseline in BPD + PTSD (0.63 ± 0.14) than in the BPD group (1.58 ± 0.22) (*t*_(55)_ = 1.71, *p* = 0.057).

Results of the multiple regression analyses are reported in online Supplementary Table S5. In BPD, no effect reached statistical significance (all *p* > 0.025). In BPD + PTSD, dissociation positively predicted HF-HRV during regulating minus attending negative pictures (*F*_(3,19)_ = 4.08, *p* = 0.025, *R*^2^ = 0.433, *R*^2^_(adjusted)_ = 0.327), with DSS-4 scores at baseline being a significant unique predictor, when controlling for dissociation at other time points (*B* = 0.812, s.e. = 0.233, *β* = 1.10, *t* = 3.49, *p* = 0.003, online Supplementary Fig. S3). Results remained significant, when controlling for arousal, STAI, ERQ, DERS, BDI, and BSL scores as covariate^3^ (online Supplementary Table S5, for correlations see online Supplementary Table S6).

## Discussion

This study investigated HF-HRV during baseline and an ER paradigm with positive, negative, and neutral pictures in 20 BPD patients with comorbid PTSD (BPD + PTSD), 37 patients without this comorbidity (BPD), and 27 HC. BPD patients, both with and without co-occurring PTSD, reported increased arousal and lower wellbeing, independent of ER instruction and picture valence. BPD + PTSD showed significantly lower HF-HRV than HC and BPD. State dissociation at baseline positively predicted HF-HRV for down-regulating (minus attending) negative pictures in BPD + PTSD.

### Subjective ratings

Altered emotional processing is a core feature of BPD. Ample studies revealed subjectively increased emotional sensitivity, reactivity, instability (Crowell *et al*., 2009; Carpenter and Trull, 2013; Santangelo *et al*., 2017), and maladaptive ER strategies (e.g. suppression) in BPD (Beblo *et al*., 2010; Salsman and Linehan, 2012). Consistent with this, patients in the present study reported more difficulties in ER (DERS), more suppression, and less cognitive reappraisal (ERQ) than HC. Partly in line with our hypothesis, BPD patients in both groups reported increased *emotional reactivity* in terms of higher arousal and lower well-being during the ER task. High aversive arousal is a basic characteristic of BPD (Stiglmayr *et al*., 2008) and those affected tend to perceive neutral (ambiguous) pictures as more arousing (Donegan *et al*., 2003; Domes *et al*., 2009; Dyck *et al*., 2009; Unoka *et al*., 2011; Daros *et al*., 2013). Moreover, there is growing evidence that altered emotion processing in BPD does not only apply to negative material but also to positive stimuli (Rüsch *et al*., 2007; Sieswerda *et al*., 2007; Hagenhoff *et al*., 2013; Thome *et al*., 2015). Extending these findings, our study revealed altered reactivity not only to negative stimuli but also to positive and neutral stimuli in BPD.

In contrast to our hypothesis, we did not observe group differences in *ER*: all groups perceived similar decreases in arousal and increases in well-being, when instructed to down-regulate emotional responses to negative pictures. Similarly, previous studies found no differences in the subjective implementation of ER strategies, such as cognitive reappraisal, distancing (Koenigsberg *et al*., 2009; Schulze *et al*., 2011), and distraction (Kuo *et al*., 2016), although BPD patients showed altered neural activity in circuits implicated in ER (Koenigsberg *et al*., 2009; Schulze *et al*., 2011; Paret *et al*., 2016; Schmitt *et al*., 2016; Zilverstand *et al*., 2017). In part, methodological aspects may explain these discrepancies, as subjective ratings generally involve the risk of conscious and/or unconscious response biases.

### HF-HRV

Despite similar self-appraisals in both BPD groups, only patients with comorbid PTSD showed significant HF-HRV alterations during the ER task. Again, group differences were independent of instruction and picture valence: HF-HRV levels were significantly lower in BPD + PTSD than in HC and (as a trend) in BPD across all conditions. When excluding medicated patients, this pattern remained significant. Extending previous research (Meyer *et al*., 2016), our findings indicate a significant impact of comorbid PTSD on HF-HRV, not only under baseline conditions but also during an ER task in BPD, pointing to complex interactions between (post)traumatic stress and autonomic nervous system functioning (Pechtel and Pizzagalli, 2011; Sammito *et al*., 2015). Other variables that may affect HF-HRV, such as depressive symptoms, BPD symptom severity, and comorbidities other than PTSD did not differ between the patient groups, rendering it unlikely that they accounted for the observed group differences in HF-HRV.

In line with previous studies applying ER tasks, we did not find evidence for significant deficits in *ER*, i.e. HF-HRV differences dependent on the ER instruction, in BPD. Likewise, patients with BPD previously showed no differences in HRV when implementing and strengthening ER skills (Svaldi *et al*., 2012; Metcalfe *et al*., 2015; Fitzpatrick and Kuo, 2016; Kuo *et al*., 2016; Dixon-Gordon *et al*., 2017).

Interestingly, state dissociation positively predicted HF-HRV during ER in BPD + PTSD. More specifically, patients with BPD + PTSD, who experienced higher dissociation, particularly at baseline, showed higher HF-HRV when instructed to down-regulate (minus passively attend) negative pictures. In line with this, BPD patients with higher dissociation previously showed reduced sympathetic reactivity, suggesting better emotional adaptation, after induction of anger and sadness (Fitzpatrick and Kuo, 2015). Moreover, dissociation was associated with dampened startle responses, SCR (Ebner-Priemer *et al*., 2005; Ebner-Priemer *et al*., 2009; Barnow *et al*., 2012) and limbic reactivity (Krause-Utz *et al*., 2017) to stressful material in BPD. At the same time, dissociation was found to interfere with learning and memory (Ebner-Priemer *et al*., 2009; Krause-Utz *et al*., 2017) and predicted poor treatment outcome (Arntz *et al*., 2015; Kleindienst *et al*., 2016). In the context of these earlier findings, our results suggest that dissociation may underlie attempts of increasing ER, possibly at the cost of other mental processes that are crucial to goal-directed behavior (Lanius *et al*., 2010). More specifically, our present findings provide preliminary evidence for an impact of dissociation on HF-HRV during ER in BPD patients with comorbid PTSD. Much more research is needed to shed more light on this relationship and how it may relate to treatment outcome. Future studies should investigate effects of experimentally induced dissociation on HF-HRV during an ER task to gain more insight into possible causal relationships.

### Limitations

To our knowledge, this is the first study in BPD examining HF-HRV during an ER task with positive and negative stimuli, taking comorbid PTSD and dissociation into account. Findings for the ER task remained stable after excluding medicated patients, rendering it unlikely that results are explained by medication status. However, exclusion of medicated patients reduced our sample sizes, restricting the statistical power to detect differential effects. As we only included women, findings cannot be generalized to male populations. Since we did not include a control group of PTSD patients without BPD, we cannot conclude whether findings are specific to PTSD or the co-occurrence of BPD + PTSD, which may worsen psychopathology. While patient groups did not differ concerning comorbidities other than PTSD, comorbidities in these groups may have affected results. Findings did not change after excluding patients with comorbid major depression. However, studies with larger subsamples of patients with and without comorbid depression are needed to replicate our findings. Moreover, the ecological validity of our ER paradigm may be limited. Future study should link HF-HRV to ER in every-day life, especially in interpersonal situations, which may be particularly challenging for individuals with BPD (Schmahl *et al*., 2014).

In conclusion, reduced HF-HRV during baseline and an ER task may be particularly linked to comorbid PTSD. Dissociation may underlie attempts to increase ER and HF-HRV in BPD patients with this comorbidity, which should be considered in future research and treatment.

## Footnotes

† The notes appear after the main text.

## Notes

1 *η*_p_^2^ ⩽ 0.039 representing small effects, *η*_p_^2^ = 0.04–0.139 mediate effects, and *η*_p_^2^ ⩾ 0.14 large effects.

2 *d* = 0.20 representing small effects, *d* = 0.50 mediate effects, and *d* = 0.80 large effects.

3 When controlling for arousal (*B* = 0.795, s.e. = 0.251, *β* = 1.08, *t* = 3.17, *p* = 0.006), STAI (*B* = 0.984, s.e. = 0.305, *β* = 1.47, *t* = 3.23, *p* = 0.006), ERQ reappraisal (*B* = 0.727, s.e. = 0.238, *β* = 0.984, *t* = 3.06, *p* = 0.008), ERQ suppression (*B* = 0.809, s.e. = 0.239, *β* = 1.10, *t* = 3.38, *p* = 0.004), DERS (*B* = 0.919, s.e. = 0.247, *β* = 1.25, *t* = 3.73, *p* = 0.002), BDI (*B* = 0.887, s.e. = 0.260, *β* = 1.20, *t* = 3.41, *p* = 0.004), and BSL (*B* = 1.01, s.e. = 0.219, *β* = 1.37, *t* = 4.60, *p* < 0.001).

## Supplementary material

For supplementary material accompanying this paper visit https://doi.org/10.1017/S0033291718002489. click here to view supplementary material
