# Supplementary material for: Heart rate variability during a cognitive reappraisal task in female patients with borderline personality disorder: the role of comorbid posttraumatic stress disorder and dissociation
Source: Psychol Med. 2018 Sep 10;49(11):1810–21. doi: 10.1017/S0033291718002489 (PMC6650777; doi:10.1017/S0033291718002489)
Supplement: Supplementary file 1 [file S0033291718002489sup001.zip › S0033291718002489sup001/Supplemental_Material.docx]

**Self-report scales of trait emotion regulation strategies**

*Difficulties in Emotion Regulation Scale (DERS) (Gratz & Roemer, 2004)*

Self-reported difficulties in emotion regulation were assessed using the DERS, a self-report questionnaire consisting of 36 items that refer to six subscales: Non-acceptance of emotions (e.g., “*When I'm upset, I feel guilty for feeling that way*”), Difficulties engaging in goal directed behaviour (e.g., “*When I'm upset, I have difficulty concentrating*”), Impulse control difficulties (e.g., “*When I'm upset, I lose control over my behaviours*”), Lack of emotional awareness (e.g., “*I am attentive to my feelings*”), Limited access to emotion regulation strategies (e.g., “*When I'm upset, I believe that I'll end up feeling very depressed*”), and Lack of emotional clarity (e.g., “*I have difficulty making sense out of my feelings*”). Each item is rated on a five-point Likert scale from 1 (almost never) to 5 (almost always). In the present study, items were scored in the way that higher scores indicate more difficulties in emotion regulation. Good internal consistency of the subscales (Cronbach’s α=.80-.89) and test-retest-reliability after 4-8 weeks (r=.88) as well as good construct validity were reported (Bardeen, Fergus, & Orcutt, 2012; Gratz & Roemer, 2004). In the present study, Cronbach’s α was 0.98 for the total scale and 0.88 - 0.95 for the subscales, indicating good to excellent internal consistency.

*Emotion Regulation Questionnaire (ERQ) (Gross & John, 2003)*

A modified version of the Emotion Regulation Questionnaire (ERQ) (Gross & John, 2003) was used to assess the two major emotion regulation strategies ‘cognitive reappraisal’ and ‘suppression’ during the past seven days. The ERQ consists of 10 items which are answered on a 7-point Likert scale (1=not at all true, 7=completely true). Six items refer to the preferred use of cognitive reappraisal as an emotion regulation strategy, while 4 items assess suppression as a preferred strategy. The internal consistency of the scale was found to be acceptable to good (cognitive reappraisal: α=.74-.82; suppression: α=.76) (Gross & John, 2003) and there is evidence for good construct validity (Wiltnik et al., 2011). In personal email communication with J. Gross (2014), the scale was slightly adapted to assess emotion regulation strategies during the last 7 days. In the present study, Cronbach’s alpha for the subscales was 0.83 (cognitive reappraisal) and 0.76 (suppression) respectively, indicating acceptable to good internal consistency.

**Code of IAPS pictures used in the ER task**

**Negative pictures**

3550, 9921, 2703, 3400, 9427, 9570, 9635, 3181, 9254, 3059, 2811, 8230, 2053, 6415, 9420, 3010, 6370, 9325, 3051, 9332, 9185, 2691, 3064, 3220, 9163, 9412, 9181, 2095, 3301, 9491, 9187, 6350, 9571, 9321, 6230, 9910, 3103, 3063, 9905, 3300, 3100, 9908

**Neutral pictures**

2383, 7026, 2890, 7001, 5635, 2025, 7019, 7041, 2495, 2200, 7002, 2411, 2499, 7032, 2594, 7513, 2480, 7010, 2305, 7003, 2357

**Positive pictures**

1460, 2035, 5830, 2058, 7330, 5200, 5820, 1710, 2331, 2050, 8185, 2151, 4610, 5001, 5210, 2209, 2340, 2311, 1811, 8190, 8200, 8496, 2045, 1750, 1604, 2530, 2216, 5480, 2341, 7502, 1500, 5910, 1590, 2154, 5833, 1340, 5831, 2332, 2057, 5825, 2071, 1999
