# Supplementary material for: Heart rate variability during a cognitive reappraisal task in female patients with borderline personality disorder: the role of comorbid posttraumatic stress disorder and dissociation
Source: Psychol Med. 2018 Sep 10;49(11):1810–21. doi: 10.1017/S0033291718002489 (PMC6650777; doi:10.1017/S0033291718002489)
Supplement: Supplementary file 1 [file S0033291718002489sup001.zip › S0033291718002489sup001/Supplemental_Table_1.docx]

Supplemental Table 1

*Complete list of psychotropic medication*

| Group | Substance (Brand name), dosage per day |
| --- | --- |
| BPD | Venlafaxine, 75 mg; Bupropion, 300 mg |
|  | Citalopram, 20mg; Quetiapin, 10 mg |
|  | Citalopram, 20mg; Mirtazapine (Remeron), 15 mg |
|  | Fluoxetine, 60 mg; Quetiapine (Seroquel), 50 mg |
|  | Escitalopram (Cipralex), 20 mg |
|  | Venlafaxine, 150 mg |
|  | Fluoxetine, 40 mg; Lamotrigine (Lamictal), 200 mg |
|  | Citalopram, 40 mg |
|  | Bupropion, 300 mg; Lyrica, 600 mg; Fluoxetine, 40 mg; Quetiapine (Seroquel), 25 mg |
|  | Sertraline, 100 mg; Quetiapine (Seroquel), 600 mg |
|  | Fluoxetin, 20mg; Trazodone, 12,5 mg |
|  | Perazine (Taxilan), 50 mg |
|  | Sertraline 50 mg |
|  | Sertraline 50 mg |
|  | Venlafaxine, 225 mg; Quetiapine (Seroquel), 12,5 mg |
| BPD_PTSD | Escitalopram (Cipralex), 30 mg; Trazodone, 200 mg |
|  | Venlafaxine, 300 mg |
|  | Venlafaxine, 225 mg |
|  | Citalopram, 20 mg |
|  | Risperidon, 1mg; Venlafaxine, 75 mg |
|  | Sertralin 50 mg, Trimipramin, Quetiapin (not daily/regularly) |
|  | Fluoxetine, 30mg |
|  | Sertraline (Zoloft), 150 mg; Prothipendyl, 40 mg |

Note: This table provides a a list of the prescribed psychotropic medication (substance, brand name, and dosage per day) for each of the 23 medicated patients in our sample. BPD=Borderline Personality Disorder, BPD_PTSD=Patients with BPD and comorbid Posttraumatic Stress Disorder
