# Supplementary material for: Heart rate variability during a cognitive reappraisal task in female patients with borderline personality disorder: the role of comorbid posttraumatic stress disorder and dissociation
Source: Psychol Med. 2018 Sep 10;49(11):1810–21. doi: 10.1017/S0033291718002489 (PMC6650777; doi:10.1017/S0033291718002489)
Supplement: Supplementary file 1 [file S0033291718002489sup001.zip › S0033291718002489sup001/Supplemental_Table_2.docx]

Supplemental Table 2:

*Results of the analyses for absolute HF-HRV values for healthy controls (n=27), patients without Borderline Personality Disorder with comorbid Posttraumatic Stress Disorder (BPD+PTSD: n=20) and BPD patients without comorbid PTSD (BPD: n=37)*

|  | *F(df)* | *P* | *η_p_²* |
| --- | --- | --- | --- |
| ***Baseline: Univariate ANOVA*** | | | |
| Group | *F*_(2,81)_=2.36 | *p*=.101 |  |
| ***Emotional Reactivity: 3x3 rm-ANOVA*** | | | |
| Group | *F*_(2,81)_=4.08, | *p*=.020, | *η_p_²*=0.09 |
| Valence | *F*_(2,81)_=1.11, | *p*=.334 |  |
| Group x valence | *F*_(2,160)_=0.82, | *p*=.517 |  |
| ***Emotional Regulation: 3x2x3 rm-ANOVA*** | | | |
| Group | *F*_(2,81)_= 4.73, | *p*=.011, | *η_p_²*=0.11 |
| Instruction | *F*_(1,81)_=3.43, | *p*=.068 | *η_p_²*=0.04 |
| Valence | *F*_(1,81)_=0.06, | *p*=.801 |  |
| Group x Instruction | *F*_(2,81)_=0.54, | *p*=.585 |  |
| Group x Valence | *F*_(2,81)_=0.40, | *p*=.672 |  |
| Valence x Instruction | *F*_(1,81)_=2.68, | *p*=.105 |  |
| Group x Valence x Instruction | *F*_(2,81)_=1.86, | *p*=.163 |  |
