# Supplementary material for: Heart rate variability during a cognitive reappraisal task in female patients with borderline personality disorder: the role of comorbid posttraumatic stress disorder and dissociation
Source: Psychol Med. 2018 Sep 10;49(11):1810–21. doi: 10.1017/S0033291718002489 (PMC6650777; doi:10.1017/S0033291718002489)
Supplement: Supplementary file 1 [file S0033291718002489sup001.zip › S0033291718002489sup001/Supplemental_Table_3.docx]

Supplemental Table 3:

*Results of the HF-HRV analyses for Borderline Personality Disorder patients without comorbid major depression (BPD+PTSD: n=17, BPD: n=28) and healthy controls (n=27)*

|  | *F(df)* | *P* | *η_p_²* |
| --- | --- | --- | --- |
| ***Baseline: Univariate ANOVA*** | | | |
| Group | *F*_(2,69)_=1.52 | *p*=.227 |  |
| ***Emotional Reactivity: 3x3 rm-ANOVA*** | | | |
| Group | *F*_(2,69)_=6.43, | *p*=.003, | *η_p_²*=0.16 |
| Valence | *F*_(2,69)_=0.62, | *p*=.536 |  |
| Group x valence | *F*_(2,69)_=1.08, | *p*=.367 |  |
| ***Emotional Regulation: 3x2x3 rm-ANOVA*** | | | |
| Group | *F*_(2,69)_= 6.97, | *p*=.002, | *η_p_²*=0.17 |
| Instruction | *F*_(1,69)_=2.64, | *p*=.109 |  |
| Valence | *F*_(1,69)_=0.10, | *p*=.323 |  |
| Group x Instruction | *F*_(2,69)_=0.54, | *p*=.585 |  |
| Group x Valence | *F*_(2,69)_=0.24, | *p*=.781 |  |
| Valence x Instruction | *F*_(1,69)_=3.45, | *p*=.068, | *η_p_²*=0.05 |
| Group x Valence x Instruction | *F*_(2,69)_=1.06, | *p*=.354 |  |
