# Supplementary material for: Heart rate variability during a cognitive reappraisal task in female patients with borderline personality disorder: the role of comorbid posttraumatic stress disorder and dissociation
Source: Psychol Med. 2018 Sep 10;49(11):1810–21. doi: 10.1017/S0033291718002489 (PMC6650777; doi:10.1017/S0033291718002489)
Supplement: Supplementary file 1 [file S0033291718002489sup001.zip › S0033291718002489sup001/Supplemental_Table_4.docx]

Supplemental Table 4a:

*Results of the HF-HRV analyses of variance with medication status as covariate*

|  | *F(df)* | *P* | *η_p_²* |
| --- | --- | --- | --- |
| ***Baseline: Univariate ANOVA*** | | | |
| Group | *F*_(2,78)_=2.10 | *p*=.130 |  |
| ***Emotional Reactivity: 3x3 rm-ANOVA*** | | | |
| Group | *F*_(2,81)_=4.92, | *p*=.010, | *η_p_²*=0.11 |
| Valence | *F*_(2,80)_=0.31, | *p*=.732 |  |
| Group x valence | *F*_(4,158)_=0.86, | *p*=.491 |  |
| ***Emotional Regulation: 3x2x3 rm-ANOVA*** | | | |
| Group | *F*_(2,80)_= 5.26, | *p*=.007, | *η_p_²*=0.12 |
| Instruction | *F*_(1,80)_=3.00, | *p*=.087 | *η_p_²*=0.04 |
| Valence | *F*_(1,80)_=2.15, | *p*=.146, |  |
| Group x Instruction | *F*_(2,80)_=0.67, | *p*=.515 |  |
| Group x Valence | *F*_(2,80)_=0.31, | *p*=.735 |  |
| Valence x Instruction | *F*_(1,80)_=1.27, | *p*=.262 |  |
| Group x Valence x Instruction | *F*_(2,81)_=1.74, | *p*=.183 |  |

Supplemental Table 4b:

*Results of the analyses of variance for HF-HRV for healthy controls (n=27) and patients without medication (BPD: n=21, BPD+PTSD: n=12)*

|  | *F(df)* | *P* | *η_p_²* |
| --- | --- | --- | --- |
| ***Baseline: Univariate ANOVA*** | | | |
| Group | *F*_(2,57)_=4.23 | *p*=.019, | *η_p_²*=.13 |
| ***Emotional Reactivity: 3x3 rm-ANOVA*** | | | |
| Group | *F*_(2,57)_=3.52, | *p*=.036, | *η_p_²*=0.11 |
| Valence | *F*_(2,56)_=0.08, | *p*=.926 |  |
| Group x valence | *F*_(4,158)_=0.30, | *p*=.878 |  |
| ***Emotional Regulation: 3x2x3 rm-ANOVA*** | | | |
| Group | *F*_(2,57)_= 4.18, | *p*=.020, | *η_p_²*=0.13 |
| Instruction | *F*_(1,57)_=3.25, | *p*=.077 | *η_p_²*=0.05 |
| Valence | *F*_(1,57)_=1.44, | *p*=.236, |  |
| Group x Instruction | *F*_(2,57)_=1.45, | *p*=.244 |  |
| Group x Valence | *F*_(2,57)_=0.03, | *p*=.969 |  |
| Valence x Instruction | *F*_(1,57)_=1.70, | *p*=.198 |  |
| Group x Valence x Instruction | *F*_(2,57)_=0.63, | *p*=.538 |  |
