# Supplementary material for: Heart rate variability during a cognitive reappraisal task in female patients with borderline personality disorder: the role of comorbid posttraumatic stress disorder and dissociation
Source: Psychol Med. 2018 Sep 10;49(11):1810–21. doi: 10.1017/S0033291718002489 (PMC6650777; doi:10.1017/S0033291718002489)
Supplement: Supplementary file 1 [file S0033291718002489sup001.zip › S0033291718002489sup001/Supplemental_Table_5.docx]

Supplemental Table 5

*Results of the multiple regression analyses predicting HF-HRV during the Emotion Regulation task from acute dissociation*

*BPD (n=37).*

| ***Emotional reactivity*** | | |  |  |  |  |
| --- | --- | --- | --- | --- | --- | --- |
| **Predictors** | **Dependent Variable** | | *F_(3,36)_* | *p* | *SE (estimate)* | *R², (R² adjusted)* |
| DSS T1  DSS T2  DSS T3 | HF-HRV *Neutral+attend*  HF-HRV *Positive+attend*  HF-HRV *Negative+attend* | | *1.19*  *0.50*  *1.01* | *.330*  *.685*  *.400* | *16.40*  *17.96*  *18.68* | *.097 (.015)*  *.043 (-.043)*  *.084 (.001)* |
|  | |  |  |  |  |  |

***Emotion regulation***

| **Predictors** | **Dependent Variable** | | *F_(3,36)_* | *p* | *SE (estimate)* | *R², (R² adjusted)* |
| --- | --- | --- | --- | --- | --- | --- |
| DSS T1  DSS T2  DSS T3 | *Positive_regulate_min_attend*  *Negative_regulate_min_attend* | | *0.29*  *0.84* | *.831*  *.485* | *14.84*  *12.56* | *.026 (-.063).*  *070 (-.014)* |
|  | |  |  |  |  |  |

BPD+PTSD (n=20)

| ***Emotional reactivity*** | | |  |  |  |  |
| --- | --- | --- | --- | --- | --- | --- |
| **Predictors** | **Dependent Variable** | | *F^(3,19)^* | *p* | *SE (estimate)* | *R², (R² adjusted)* |
| DSS T1  DSS T2  DSS T3 | HF-HRV *Neutral+attend*  HF-HRV *Positive+attend*  HF-HRV *Negative+attend* | | *0.18*  *0.13*  *0.19* | *.908*  *.940*  *.906* | *20.94*  *18.52*  *22.48* | *.033 (-.149)*  *.024 (-.159)*  *.033 (-.148)* |
|  | |  |  |  |  |  |

***Emotion regulation***

| **Predictors** | **Dependent Variable** | | *F_(3,19)_* | *p* | *SE (estimate)* | *R², (R² adjusted)* |
| --- | --- | --- | --- | --- | --- | --- |
| DSS T1  DSS T2  DSS T3 | *Positive_regulate_min_attend*  *Negative_regulate_min_attend* | | *0.61*  *4.08* | *.614*  *.025* ^ǂ^* | *14.52*  *9.32* | *.104 (-.064)*  *.433 (.327)* |
|  | |  |  |  |  |  |

Note: HF-HRF values are presented for the instruction to passively view pictures (*Neutral+attend, Positive+attend, Negative+attend*) and for the contrasts between the two ER conditions, regulate minus attend (*Positive_regulate_min_attend*, *Negative_regulate_min_attend*). DSS T1= Scores of the Dissociation Stress Scale 4 (DSS-4) assessed at baseline, DSS T2= Scores of the DSS-4 assessed before the task, DSS T3= Scores of the DSS-4 assessed after the task.

* significant at *p*≤.025, two-tailed.

*^ǂ^* Results remained significant, when controlling for arousal (B=.795, SE=.251, beta=1.08, *t*=3.17, *p*=.006), STAI (B=.984, SE=.305, beta=1.47, *t*=3.23, *p*=.006), ERQ reappraisal (B=.727, SE=.238, beta=.984, *t*=3.06, *p*=.008), ERQ suppression (B=.809, SE=.239, beta=1.10, *t*=3.38, *p*=.004), DERS (B=.919, SE=.247, beta=1.25, *t*=3.73, *p*=.002), BDI (B=.887, SE=.260, beta=1.20, *t*=3.41, *p*=.004), and BSL (B=1.01, SE=.219, beta=1.37, *t*=4.60, *p*<.001) scores.
