# Supplementary material for: Heart rate variability during a cognitive reappraisal task in female patients with borderline personality disorder: the role of comorbid posttraumatic stress disorder and dissociation
Source: Psychol Med. 2018 Sep 10;49(11):1810–21. doi: 10.1017/S0033291718002489 (PMC6650777; doi:10.1017/S0033291718002489)
Supplement: Supplementary file 1 [file S0033291718002489sup001.zip › S0033291718002489sup001/Supplemental_Table_6.docx]

Supplemental Table 6

*Correlations Between Scores on the DSS4 with other clinical measures*

BPD (n=37)

| Variables | 1 | 2 | 3 | 4 | 5 | 6 | 7 | 8 | 9 | 10 | 11 | 12 |
| --- | --- | --- | --- | --- | --- | --- | --- | --- | --- | --- | --- | --- |
| 1. DSS4 baseline 2. DSS4 before task 3. DSS4 after task 4. DERS total 5. ERQ suppression 6. ERQ reappraisal 7. STAI | - | .827***  - | .752***  .828***  - | .113  .035  -.105  - | .098  .217  .333*  -.554***  - | .211  .173  .164  .231  .080  - | .419*  .196  .017  .493***  -.101  .274  - | .251  .257  -.029  .633***  -.261  .178  .607*** | .404*  .369*  .130  .620***  -.233  .037  .578*** | .248  .014  -.254  .428***  -.196  .117  .751*** | .298  .307  .287  -.023  .263  .249  .248 | .271  .248  .235  .300  .043  .146  .307 |
| 1. BDI |  |  |  |  |  |  |  | - | .778*** | .403 | .032 | .258 |
| 1. BSL23 |  |  |  |  |  |  |  |  | - | .471** | .080 | .407*** |
| 1. Arousal baseline |  |  |  |  |  |  |  |  |  | - | .195 | .331* |
| 1. Arousal before task 2. Arousal after task |  |  |  |  |  |  |  |  |  |  | - | .463**  - |

Note. This table shows Pearson correlations between all variables. * p<0.05. **p<0.01; ***p<0.001. BPD=Borderline Personality Disorder, DSS4=Dissociation Stress Scale 4, DERS= Difficulties in Emotion Regulation Scale, ERQ=Emotion Regulation Questionnaire, STAI=State Trait Anxiety Inventory, state version, BDI=Beck Depression Inventory 2, BSL23=Borderline Symptom List 23.

BPD PTSD (n=20)

| Variables | 1 | 2 | 3 | 4 | 5 | 6 | 7 | 8 | 9 | 10 | 11 | 12 |
| --- | --- | --- | --- | --- | --- | --- | --- | --- | --- | --- | --- | --- |
| 1. DSS4 baseline 2. DSS4 before task 3. DSS4 after task 4. DERS total 5. ERQ suppression 6. ERQ reappraisal 7. STAI | - | .782***  - | .614***  .889***  - | .155  -.079  -.073  - | -.069  .141  .161  -.502*  - | .050  .023  -.013  -.098  .119  - | .518*  .134  -.009  .355  -.217  .332  - | .295  .035  -.026  .625**  -.279  .480*  .653** | .464*  .407  .481*  .389  -.002  .318  .367 | .362  .166  .147  -.050  .020  .458*  .670** | .065  .131  .276  -.051  -.102  .061  .247 | .203  .064  .144  .317  -.327  .147  .307 |
| 1. BDI |  |  |  |  |  |  |  | - | .490* | .313 | .114 | .457* |
| 1. BSL23 |  |  |  |  |  |  |  |  | - | .472* | .089 | .550** |
| 1. Arousal baseline |  |  |  |  |  |  |  |  |  | - | .565** | .569** |
| 1. Arousal before task 2. Arousal after task |  |  |  |  |  |  |  |  |  |  | - | .615**  - |

Note. This table shows Pearson correlations between all variables. * p<0.05. **p<0.01; ***p<0.001. BPD=Borderline Personality Disorder, PTSD=(comorbid) Posttraumatic Stress Disorder, DSS4=Dissociation Stress Scale 4, DERS= Difficulties in Emotion Regulation Scale, ERQ=Emotion Regulation Questionnaire, STAI=State Trait Anxiety Inventory, state version, BDI=Beck Depression Inventory 2, BSL23=Borderline Symptom List 23.
